# Gentamicin Targeting Human Hemoglobin Induces Methemoglobin Formation and Decreases Oxygen Affinity: A Molecular Mechanism of Hematologic Toxicity

**DOI:** 10.3390/ijms27177760

**Published:** 2026-08-29

**Authors:** Peilin Shu, Pengfei Wang, Wencong Li, Baichuan Gu, Yuanjing Zheng, Ying Wang, Minghao Yang, Lian Zhao

**Affiliations:** 1 Air Force Medical Center, Air Force Medical University, Beijing 100142, China; 18810046967@163.com (P.S.); wangpf2016@fmmu.edu.cn (P.W.); li948196130@163.com (W.L.); 18701679510@163.com (Y.Z.); 2 School of Integrated Circuits, Beijing University of Technology, Beijing 100124, China; 17828841232@163.com; 3 Academy of Military Medical Sciences, No. 27 Taiping Road, Haidian, Beijing 100850, China; wy830111@163.com

**Keywords:** Gentamicin, hemoglobin, methemoglobin, hematotoxicity, oxygen affinity, drug safety

## Abstract

The nephrotoxicity and ototoxicity of Gentamicin have been extensively investigated; however, whether they induce hematotoxicity remains unclear. This study aimed to explore the binding of Gentamicin to adult hemoglobin (HbA) and its toxicological implications. The effect of Gentamicin on HbA oxidation was assessed by quantifying methemoglobin (MetHb) formation via a four-wavelength spectrophotometric method, and scavenger rescue assays were employed to elucidate the oxidative mechanism. Alterations in the oxygen-carrying capacity of both HbA and RBCs were evaluated through the acquisition of oxygen equilibrium curves and oxygen dissociation assays. The binding affinity between Gentamicin and HbA was determined by surface plasmon resonance (SPR). Furthermore, the influence of Gentamicin on the secondary and tertiary structures of HbA was examined by microfluidic modulation spectroscopy (MMS) and UV–visible absorption spectroscopy, respectively. Molecular docking was employed to predict the binding sites of Gentamicin on HbA. Molecular dynamics simulations verified the stability of the binding. Gentamicin promoted HbA autoxidation, elevating MetHb levels, and reduced the oxygen affinity of HbA. Gentamicin-promoted HbA autoxidation is primarily mediated by both direct heme-pocket perturbation and H_2_O_2_/iron-dependent amplification. SPR confirmed concentration-dependent specific binding between Gentamicin and HbA. MMS indicated no alteration in HbA secondary structure; however, UV–visible spectroscopy revealed that Gentamicin attenuated the Soret band, converted the oxyhemoglobin double-peak to a singlet, and generated a new band at 630 nm. Molecular docking predicted that Gentamicin binds β-chain residues via hydrogen, carbon–hydrogen, and hydrophobic interactions. This study reveals that Gentamicin exerts hematological effects in vitro. By binding to specific sites on HbA, Gentamicin affects the tertiary structure of HbA, promotes heme oxidation, ultimately increases MetHb content (oxidative damage), and decreases its oxygen-carrying capacity (functional impairment).

## 1. Introduction

Gentamicin, a broad-spectrum aminoglycoside antibiotic, is employed primarily for the management of severe Gram-negative bacterial infections, including neonatal sepsis, mycobacterial infections, and meningitis [1,2]. Despite the continued emergence of novel antimicrobial agents, Gentamicin retains substantial clinical utility owing to its high potency, rapid bactericidal activity, low cost, and the advantage of circumventing broader-spectrum antibiotic use, thereby mitigating the risk of resistance development [3,4,5].

However, the therapeutic application of Gentamicin is constrained by dose-limiting toxicities, among which nephrotoxicity and ototoxicity are the most prominent [3,6]. The incidence of nephrotoxicity associated with Gentamicin therapy ranges from 10% to 25%, whereas aminoglycoside-induced hearing impairment occurs in up to 20–63% of patients receiving multiple-day dosing regimens [7,8,9,10,11].

These toxicological manifestations have been extensively investigated, and oxidative stress is recognized as a central mechanistic pathway underlying Gentamicin-induced cellular injury [6,12]. Gentamicin disrupts mitochondrial integrity, augments the generation of reactive oxygen species (ROS)—including superoxide anion and hydrogen peroxide—and promotes the liberation of catalytic iron, thereby facilitating the formation of hydroxyl radicals via the Fenton reaction. This iron-catalyzed process, operating within the Haber–Weiss cycle, is considered the principal driver of cellular damage in both renal tubular epithelia and cochlear hair cells [13,14]. Several molecular targets of Gentamicin have been identified in renal and cochlear tissues. In renal proximal tubular cells, Gentamicin binds to the endocytic receptor megalin, facilitating intracellular accumulation, and interacts with mitochondrial membranes, directly impairing electron transport chain function [15]. In cochlear hair cells, Gentamicin targets the mechanotransduction channel complex and the mitochondrial permeability transition pore, triggering apoptotic cascades [16]. Despite these advances in understanding Gentamicin organ-specific toxicity, its direct interaction with circulating blood components—particularly hemoglobin within erythrocytes—remains entirely unknown.

This knowledge gap is significant because hemoglobin, the oxygen-carrying protein of erythrocytes, depends on heme iron remaining in the ferrous (Fe^2+^) state for its oxygen-binding function [17]. Oxidation of heme iron to the ferric (Fe^3+^) state yields methemoglobin, a derivative incapable of reversible oxygen binding, thereby compromising tissue oxygen delivery [18,19]. Furthermore, hemoglobin’s oxygen supply efficiency is governed by its oxygen affinity, quantified by P50—the partial pressure of oxygen at 50% saturation on the oxygen dissociation curve—cooperativity, expressed as the Hill coefficient, and the Bohr effect, which describes pH-dependent modulation of oxygen affinity [20,21]. The theoretical oxygen release capacity (ΔSO_2_), reflecting the ability of blood to deliver oxygen under varying physiological conditions, is illustrated in the Supplementary Materials (Figure S1).

Although certain antibiotics, such as oxytetracycline and doxorubicin, have been reported to induce methemoglobin formation through direct binding or oxidative modification [22,23,24], whether Gentamicin directly interacts with hemoglobin to compromise its structural integrity and function remains entirely unexplored.

Accordingly, the present study aimed to: (1) investigate the effect of Gentamicin on MetHb formation in adult hemoglobin (HbA); (2) assess whether Gentamicin exposure alters the oxygen supply efficiency of HbA and adult RBCs; and (3) elucidate the underlying oxidative and binding mechanisms.

## 2. Results

### 2.1. Gentamicin Promotes HbA Autoxidation Through H_2_O_2_-Dependent, Iron-Mediated Oxidative Chemistry

Antioxidant activity was assessed by quantifying MetHb formation in HbA and the HbA–Gentamicin complex.

**Figure 1:**
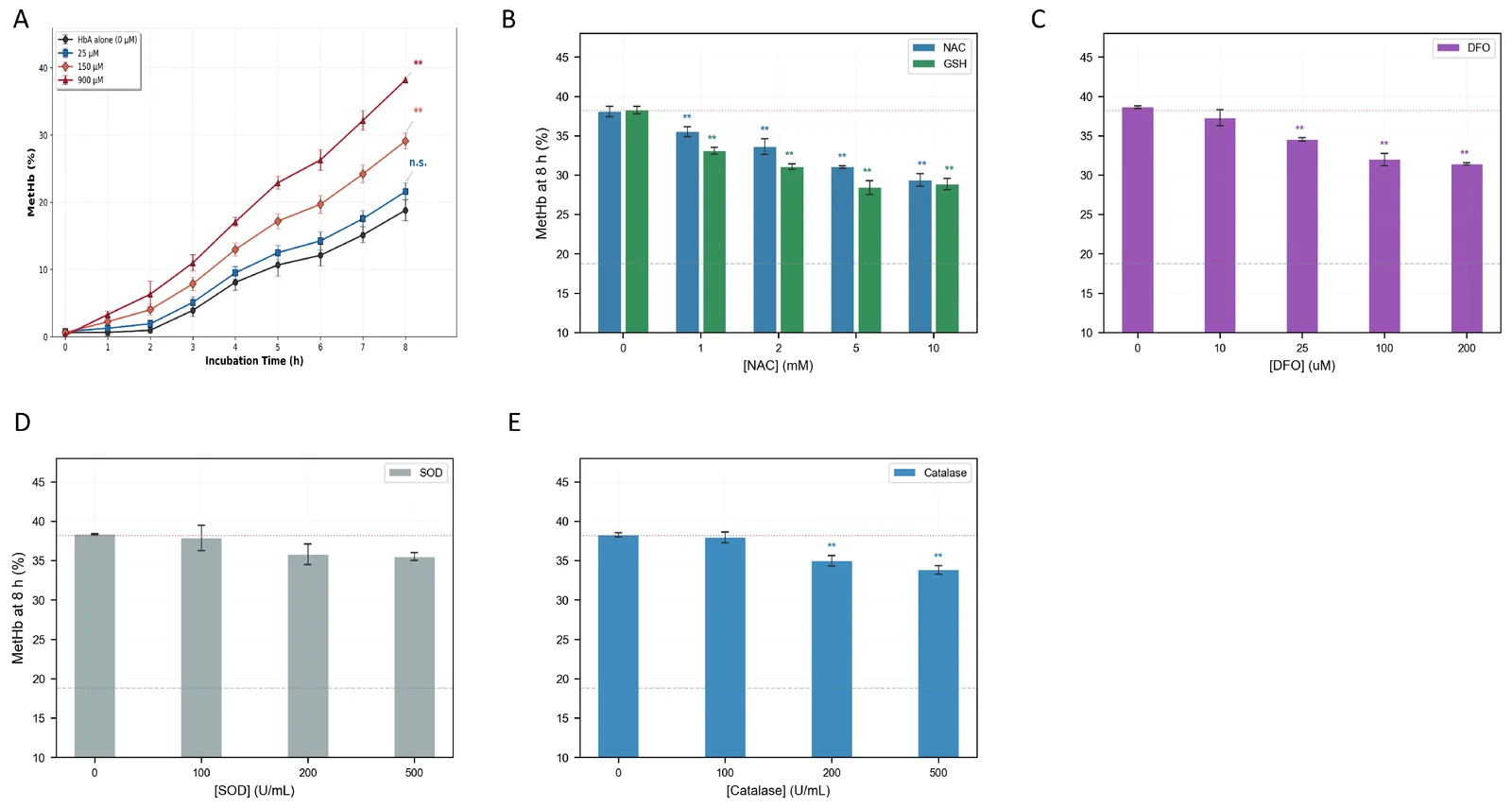
Gentamicin-induced methemoglobin formation and scavenger rescue: (**A**) Time course of MetHb formation in HbA incubated with 0, 25, 150, or 900 μM Gentamicin for 8 h. (**B**–**E**) MetHb levels at 8 h in the presence of 900 μM Gentamicin plus the indicated concentrations of NAC/GSH (**B**), DFO (**C**), SOD (**D**), or Catalase (**E**). ** *p* < 0.01 vs. 0 concentration. n.s., not significant.

Figure 1A shows the time course of MetHb concentration. Gentamicin promoted MetHb formation in a concentration-dependent manner. At the 8 h endpoint, MetHb levels were 18.77 ± 0.54% (0 μM, HbA alone), 21.39 ± 1.48% (25 μM, *p* > 0.05), 29.33 ± 0.76% (150 μM, *p* < 0.05), and 38.23 ± 0.50% (900 μM, *p* < 0.05). The dose–response relationship was monotonic across the entire concentration range tested.

To investigate whether Gentamicin-induced hemoglobin oxidation is mediated by reactive oxygen species (ROS), a series of scavenger rescue experiments were performed at a fixed pro-oxidant concentration of Gentamicin (900 μM) following 8 h of co-incubation with HbA.

The results for NAC and GSH are shown in Figure 1B. NAC decreased MetHb in a concentration-dependent manner. A significant effect was already observed at 1 mM (35.50 ± 0.62%, *p* < 0.01), and at 10 mM, MetHb was reduced from 38.23 ± 0.50% to 29.39 ± 0.81% (*p* < 0.01). Similarly, GSH exhibited significant protection at 1 mM (33.08 ± 0.41%, *p* < 0.01), lowering MetHb to 28.85 ± 0.72% at 10 mM (*p* < 0.01). These results indicate that thiol-based antioxidants effectively suppress Gentamicin-mediated HbA oxidation.

The iron chelator deferoxamine (DFO) also displayed a concentration-dependent protective effect (Figure 1C). MetHb levels were reduced to 34.52 ± 0.21% at 25 μM (*p* < 0.01), 31.97 ± 0.79% at 100 μM (*p* < 0.01), and 31.42 ± 0.15% at 200 μM (*p* < 0.01). This rescue profile suggests that free or loosely bound iron participates in a Fenton-like reaction during Gentamicin-induced HbA oxidation.

To identify the specific ROS involved, the effects of superoxide dismutase (SOD) and catalase were further examined (Figure 1D,E). SOD failed to rescue MetHb formation, with MetHb levels of 37.87 ± 1.61%, 35.78 ± 1.32%, and 35.49 ± 0.50% across the tested concentrations, none of which differed significantly from the control (38.32 ± 0.09%). In contrast, catalase significantly—though partially—reduced MetHb levels at 200 U/mL (34.95 ± 0.67%, *p* < 0.01) and 500 U/mL (33.80 ± 0.53%, *p* < 0.01) (Figure 1E).

Taken together, the differential rescue profile—protection by NAC, GSH, DFO, and catalase, but not by SOD—indicates that hydrogen peroxide and iron-dependent oxidative chemistry, rather than superoxide anion, are the primary mediators of Gentamicin-promoted HbA autoxidation.

### 2.2. Gentamicin Reduces the Oxygen-Carrying Capacity of HbA and RBCs

**Figure 2:**
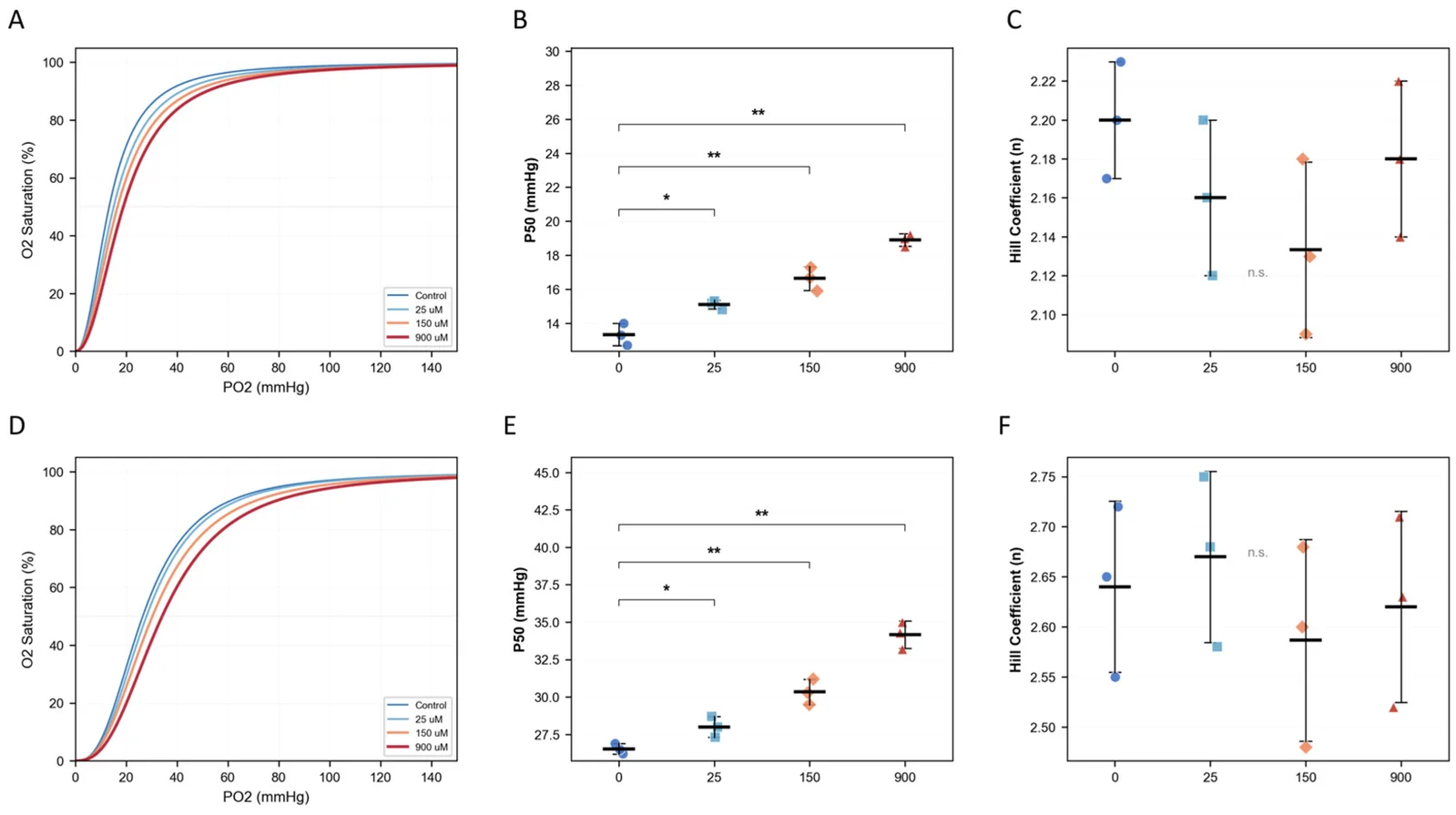
Concentration-dependent effect of Gentamicin on the oxygen affinity of HbA and adult human RBCs: (**A**) The ODC of HbA and HbA–Gentamicin. (**B**) P50 value of HbA and HbA–Gentamicin. Gentamicin significantly increased the P50 value of HbA. (**C**) Hill coefficient of HbA and HbA–Gentamicin. (**D**) The ODC of adult human RBC–Gentamicin. (**E**) P50 value of RBC and RBC–Gentamicin. Gentamicin significantly increased the P50 value of RBC. (**F**) Hill coefficient of RBC and RBC–Gentamicin. Error bars represent the standard deviation (SD). * *p* < 0.05. ** indicates *p* < 0.01. n.s., not significant.

ODC were recorded. As shown in Figure 2. the P50 of HbA was 13.33 ± 0.65 mmHg (0 μM), which increased to 15.10 ± 0.26 mmHg at 25 μM (*p* < 0.05), 16.63 ± 0.70 mmHg at 150 μM (*p* < 0.01), and 18.90 ± 0.36 mmHg at 900 μM (*p* < 0.05). For adult human RBCs, the P50 was 26.57 ± 0.60 mmHg (0 μM), and Gentamicin elevated it to 29.00 ± 1.11 mmHg at 25 μM (*p* < 0.05), 31.40 ± 0.90 mmHg at 150 μM (*p* < 0.01), and 34.27 ± 0.91 mmHg at 900 μM (*p* < 0.01). These data demonstrate that Gentamicin decreased the oxygen affinity of HbA and adult human RBCs in a concentration-dependent manner. Additionally, Gentamicin did not significantly alter the Hill coefficient in either system (*p* > 0.05 for all comparisons). The effects of Gentamicin on the oxygen-carrying capacity of rat RBCs are shown in Supplementary Materials (Section S4). Red blood cell integrity was verified by measuring free hemoglobin and LDH release; detailed procedures are provided in the Supplementary Materials (Section S5).

### 2.3. Gentamicin Has No Impact on the Bohr Effect of HbA

**Figure 3:**
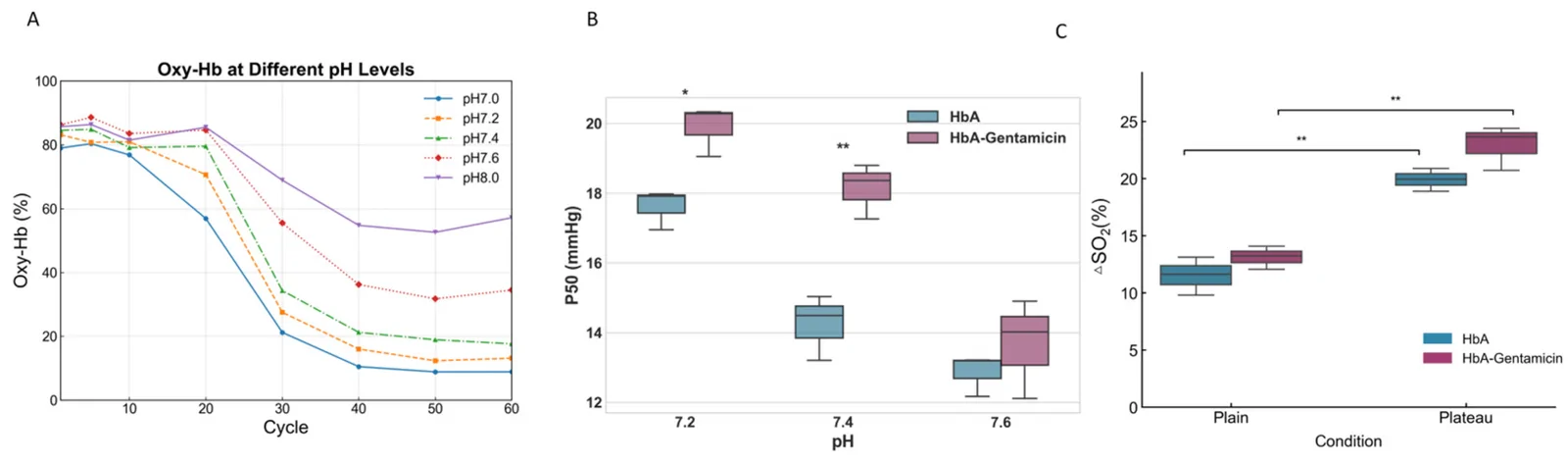
Effect of Gentamicin on the Bohr Effect of HbA: (**A**) ODA recordings for HbA and the HbA–Gentamicin complex at different pH values over 50 deoxygenation cycles. (**B**) Effects of Gentamicin on P50 of HbA at pH 7.2, 7.4, and 7.6. (**C**) Gentamicin exerts no significant effect on the theoretical oxygen-release capacity of HbA in plain or plateau environments. Error bars represent the standard deviation (SD). ** indicates *p* < 0.01.* indicates *p* < 0.05.

Figure 3A presents the ODA results for the HbA–Gentamicin complex under different pH conditions. As the pH increased, the Oxy-Hb% declined more slowly, indicating that the complex retained a normal Bohr effect. Figure 3B compares the P50 value of HbA and the HbA–Gentamicin complex at pH 7.2, 7.4, and 7.6. At pH 7.2, the P50 of the complex was significantly higher than that of HbA alone (19.89 ± 0.73 mmHg vs. 17.61 ± 0.57 mmHg, *p* < 0.05). At pH 7.4, the P50 of the complex also exceeded that of HbA (18.14 ± 0.79 mmHg vs. 14.24 ± 0.94 mmHg, *p* < 0.01). Although the P50 of the HbA–Gentamicin complex remained higher than that of HbA at pH values tested, it decreased with increasing pH, a finding that is in agreement with the ODA results presented in Figure 3A. Oxygen release was further quantified for both groups, as shown in Figure 3C. Gentamicin exerted no significant effect on the theoretical oxygen-release capacity of HbA in either a plain environment (HbA–Gentamicin: 14.62% ± 0.50% vs. HbA: 11.21% ± 1.72%) or a plateau environment (HbA–Gentamicin: 24.55% ± 0.25% vs. HbA: 19.40% ± 1.33%).

### 2.4. Identification of the Binding Between Gentamicin and HbA

**Figure 4:**
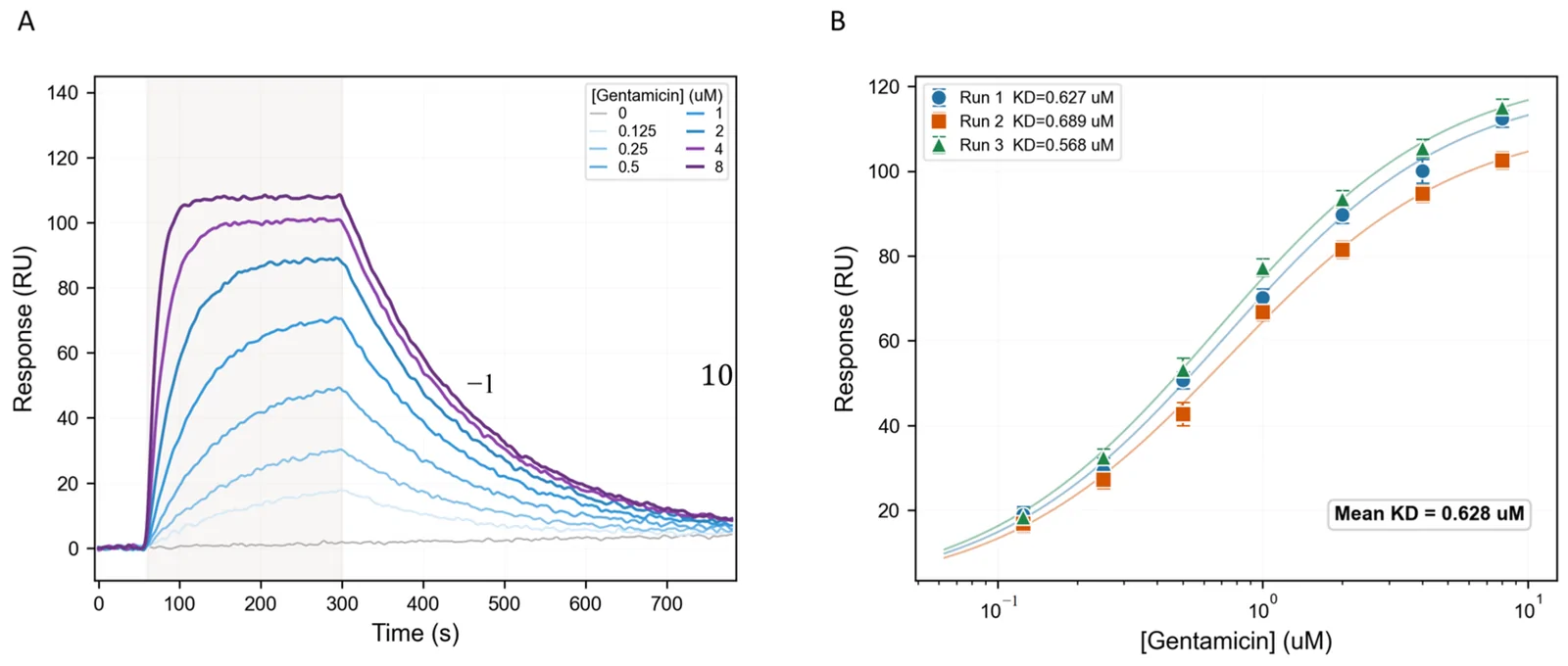
Gentamicin bound to HbA in a dose-dependent manner in the SPR assay: (**A**) Representative SPR sensorgrams (Run 1) at 0–8 μM Gentamicin. (**B**) Steady-state binding isotherm (three independent runs).

Figure 4A presents the multi-concentration SPR sensorgrams for Gentamicin binding to HbA. The binding was concentration-dependent: as the Gentamicin concentration increased from 0 to 8 μM, the SPR response rose progressively. The association rate constant (Ka), dissociation rate constant (Kd), and equilibrium dissociation constant (KD) for the Gentamicin–HbA interaction are listed in Table 1.

The steady-state binding isotherms from three independent experiments are presented in Figure 4B. The KD values derived from steady-state fitting were 0.627 μM (Run 1), 0.689 μM (Run 2), and 0.568 μM (Run 3), yielding a mean of 0.628 μM. The small inter-run variation confirms the robustness of the SPR assay and the reproducibility of the Gentamicin–HbA binding affinity.

The fitting quality metrics were consistent across all runs: reduced chi-square ~2.0, RMSE ~2.1 RU, and all U-values > 1 (Supplementary Materials Section S3).

### 2.5. Gentamicin Does Not Disrupt the Secondary Structure of HbA

**Figure 5:**
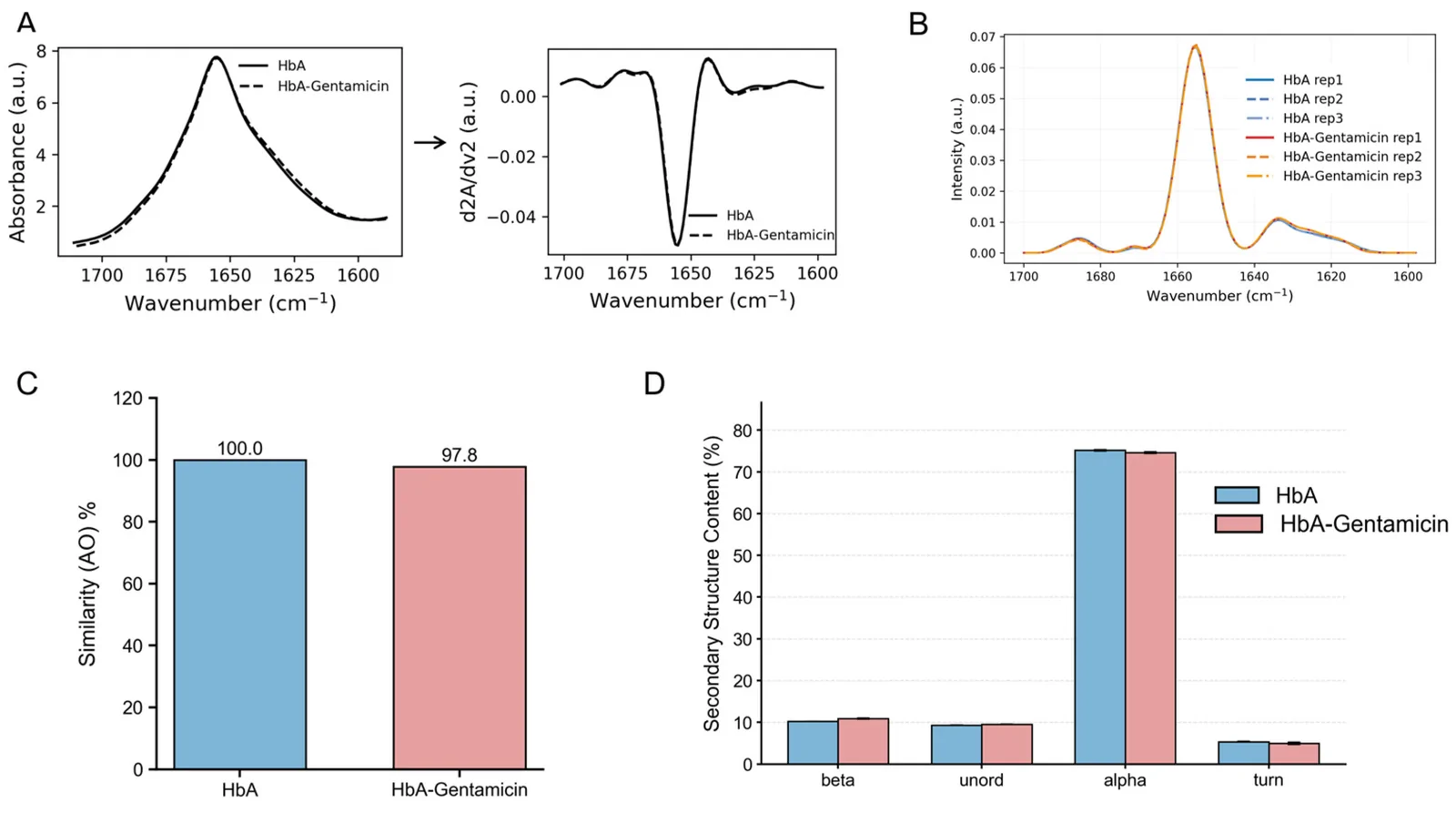
Secondary structure of HbA and HbA–Gentamicin: (**A**) Absolute absorption spectra (**left**) are converted to second derivatives (**right**). (**B**) The similarity score of the three-time experiment was tested, with similarity values of 99.75% and 99.70%, respectively. These results indicate that the experimental system was stable and the findings are reliable. (**C**) The secondary structure of the HbA–Gentamicin complex is slightly different from that of HbA. (**D**) Gentamicin has no influence on the HOS of HbA.

Figure 5A shows the absolute absorption spectrum and the corresponding second-derivative spectrum of HbA in the 1701–1598 cm^−1^ region. The strongest absorption was observed at 1652 cm^−1^, indicating that both HbA and the HbA–Gentamicin complex adopt a predominantly α-helical conformation [20]. As shown in Figure 5B,C, no statistically significant differences were observed in any secondary structure component. Specifically, α-helix content was 75.16% ± 0.16% for HbA vs. 74.62% ± 0.24% for the HbA–Gentamicin complex (*p* > 0.05). Similarly, β-sheet (10.24% ± 0.02% vs. 10.91% ± 0.14%), random coil (9.27% ± 0.06% vs. 9.51% ± 0.06%), and turns (5.33% ± 0.11% vs. 4.93% ± 0.30%) showed no significant differences (*p* > 0.05 for all) (Figure 5D).

### 2.6. Effect of Gentamicin on the Tertiary Structure of HbA

**Figure 6:**
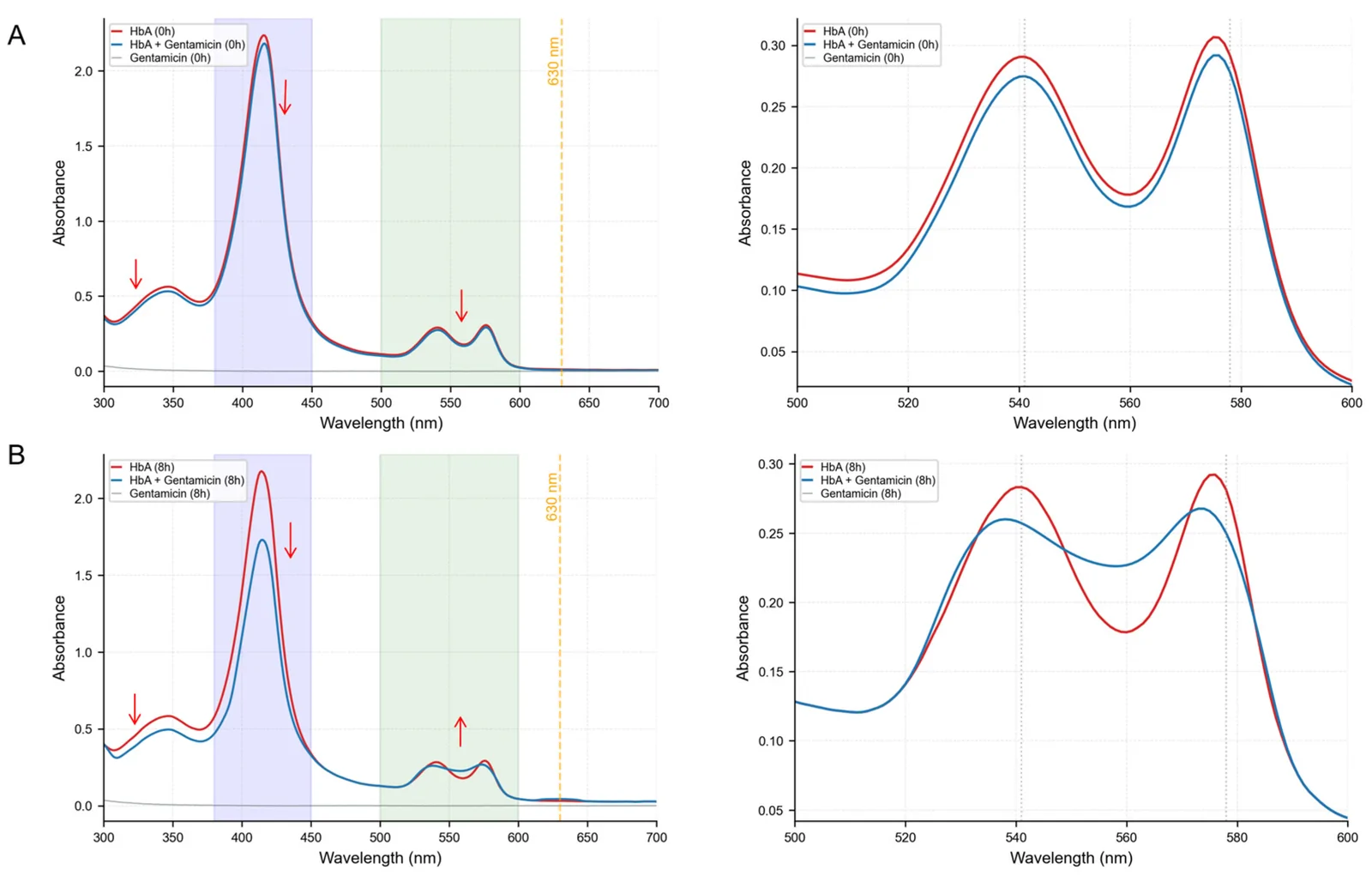
The UV-Vis spectra of HbA–Gentamicin. (**A**) UV-Vis spectra of HbA and HbA–Gentamicin at 0 h. The Q band in the left figure is zoomed in as depicted in the right figure. At 0 h, Gentamicin induces a hypochromic effect on the Q band of HbA. The red arrows indicate the changes in the globin band, S-band, and Q-band of HbA upon the addition of Gentamicin. (**B**) UV-Vis spectra of HbA and HbA–Gentamicin after 8 h. The Q band in the left figure is magnified as shown in the right figure. After 8 h, the characteristic doublet of oxyhemoglobin (at ~541/578 nm) was replaced by a single peak (at ~543–556 nm). The red arrows indicate the changes in the globin band, S-band, and Q-band of HbA upon the addition of Gentamicin. Grey lines represent the spectra of Gentamicin.

Figure 6A displays the UV–Vis spectra of HbA and the HbA–Gentamicin at 37 °C at 0 h of incubation. Upon addition of Gentamicin, the globin band (280 nm), Soret band (412 nm), and Q band (550–600 nm) of HbA all exhibited a hypochromic effect, indicating that Gentamicin can perturb the tertiary structure of HbA [25]. Gentamicin exhibited negligible absorbance (<0.001 AU) across 350–700 nm, confirming that the drug does not interfere with the spectrophotometric quantification of HbA, OxyHb, or MetHb. Figure 6B shows the UV–Vis spectra of HbA after incubation with Gentamicin for 8 h. As shown, Gentamicin caused a decrease in Soret band intensity (hypochromic effect). In the visible region, the characteristic doublet of oxyhemoglobin (at ~541/578 nm) was replaced by a single peak (at ~543–556 nm), and a new characteristic absorption band emerged at 630 nm.

### 2.7. Gentamicin Binds to HbA at βHis63, βHis92, βPhe41, βPhe103, βVal67 and βLeu141

The redocked pose of 2,3-DPG was superimposed onto the co-crystallized ligand in the original crystal structure, and the root-mean-square deviation (RMSD) for all non-hydrogen atoms was calculated to be 1.106 Å. This value is well below the widely accepted validation threshold of 2.0 Å, confirming that our docking protocol can faithfully reproduce the experimental binding mode.

**Figure 7:**
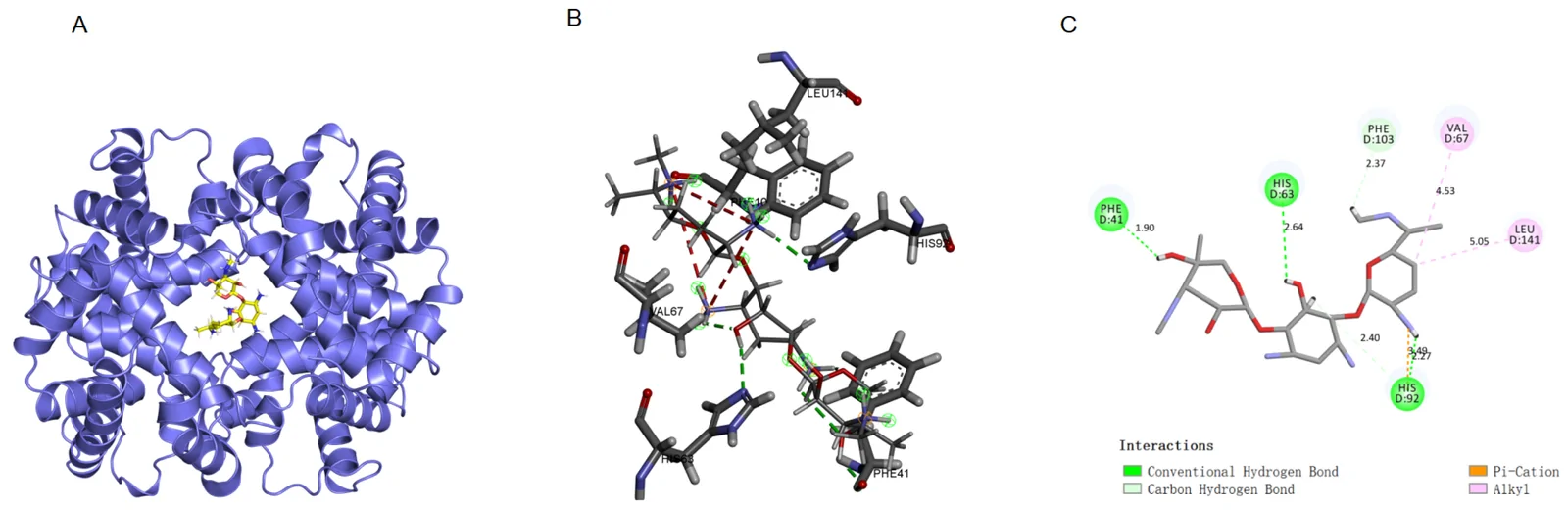
Specific binding pattern of Gentamicin to HbA: (**A**) Binding pattern of Gentamicin to HbA. Gentamicin is located in the central water cavity of HbA; HbA: red, cartoon pattern; Gentamicin: silver gray, rod. (**B**) 3D image of Gentamicin interaction with HbA. (**C**) 2D image of Gentamicin interaction with HbA.

Molecular docking predicted the most favorable ligand conformations at the active site [26], and the lowest-energy pose was selected for analysis. Gentamicin flexibly fit into the active pocket of HbA, with a CDOCKER_ENERGY of 27.0244 kJ/mol, and bound to the central hydrophobic cavity (Figure 7A). The 3D and 2D interaction schematics are shown in Figure 7B and Figure 7C, respectively. Gentamicin formed hydrogen bonds with β-chain His63, His92, and Phe41, a carbon–hydrogen bond with Phe103, and hydrophobic interactions with Val67 and Leu141.

### 2.8. Stable Binding of Gentamicin to HbA

**Figure 8:**
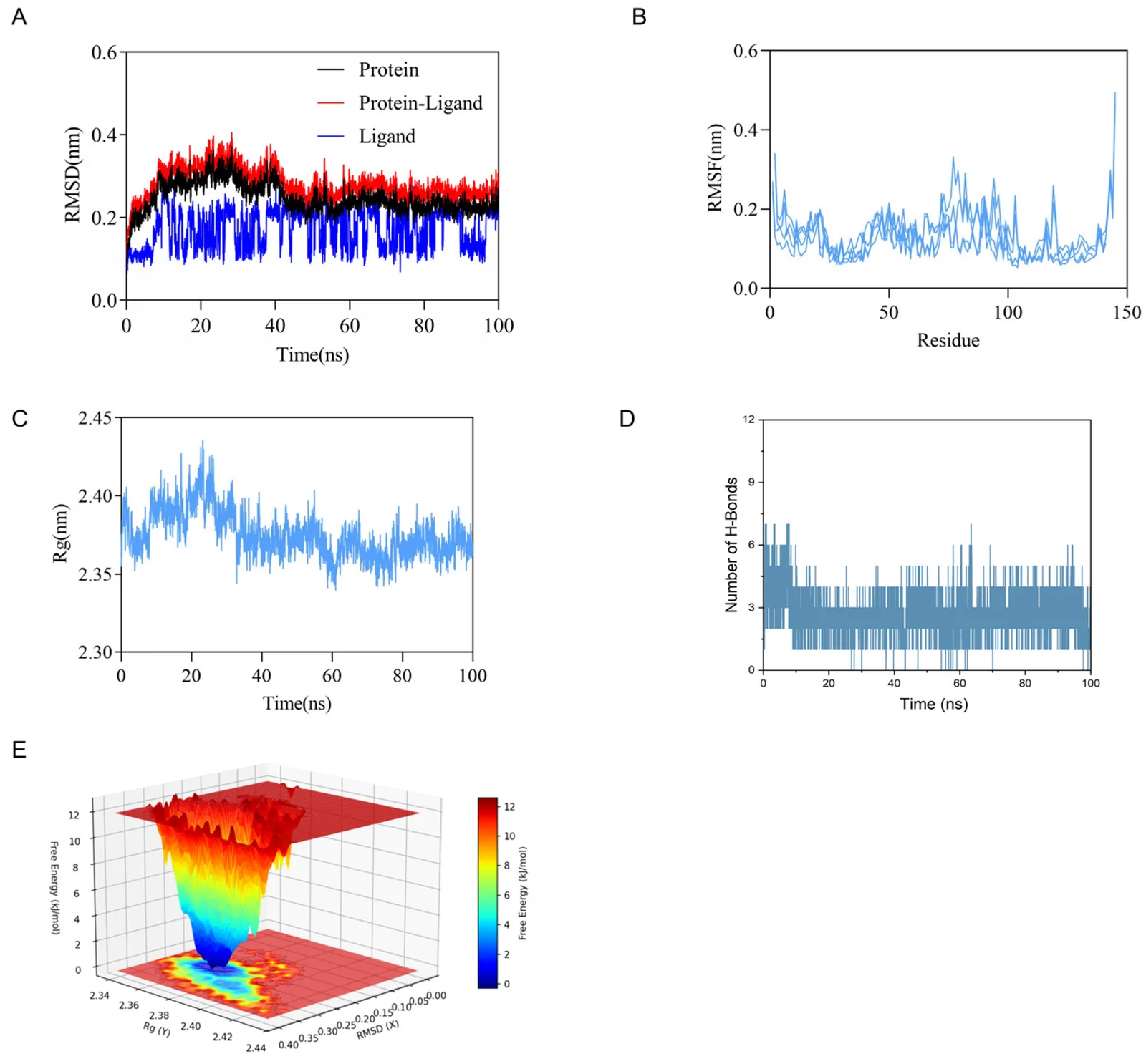
MD simulation results of the Gentamicin–HbA complex over 100 ns: (**A**) RMSD. Black: backbone of free hemoglobin; red: backbone of the protein–ligand complex; blue: Gentamicin. (**B**) RMSF profile of amino acid residues in the protein–ligand complex. Residue number is indicated on the x-axis. (**C**) Time evolution of the Rg of the protein–ligand complex. (**D**) Time course of the instantaneous number of hydrogen bonds between the protein and the ligand during the simulation. (**E**) Two-dimensional FEL constructed using RMSD and Rg as reaction coordinates. The color scale indicates free energy (kJ mol^−1^), and the blue region corresponds to the thermodynamically most stable conformational basin.

To investigate the effect of Gentamicin binding on hemoglobin conformation, 100 ns all-atom molecular dynamics (MD) simulations were performed on the free protein, the protein–ligand complex, and the free ligand. Structural stability, residue flexibility, compactness, hydrogen bonding, and free energy landscape were analyzed. The results are presented in Figure 8.

The root-mean-square deviation (RMSD) of the protein–ligand complex plateaued after 40 ns, indicating that the system had reached equilibrium. The ligand RMSD remained consistently low, indicating no dissociation. Root-mean-square fluctuation (RMSF) analysis revealed rigid core regions with natural flexibility limited to the C-termini. The radius of gyration (Rg) decreased from ~2.40 nm to a stable range of 2.37 nm. Protein–ligand hydrogen bonds were stably maintained throughout the simulation. The free energy landscape displayed a single broad low-energy basin without significant barriers. The Molecular Mechanics Poisson–Boltzmann Surface Area (MM-PBSA) binding free energy was calculated to be −69.85 kcal/mol (Table 2), supporting spontaneous and thermodynamically favorable binding.

## 3. Discussion

In summary, this study reveals that Gentamicin binds to HbA in a concentration-dependent manner and compromises its oxygen-carrying function in vitro. While the pro-oxidant effects of Gentamicin in renal and cochlear tissues are well established, our findings extend its recognized pharmacological spectrum to the circulatory system and identify HbA as a direct molecular target.

### 3.1. Gentamicin Promotes Methemoglobin Formation

Our results demonstrate that Gentamicin promotes MetHb formation, a finding consistent with its documented pro-oxidant activity. Gentamicin -induced acute kidney injury is primarily attributed to oxidative stress in renal cortical mitochondria. Likewise, in the inner ear, Gentamicin triggers the generation of free radicals, leading to irreversible damage to sensory cells and neurons [27].

Scavenger experiments dissected the chemical origin of this oxidation. NAC and GSH reduced MetHb formation by approximately 42% and 47%, respectively, confirming that a substantial fraction of the damage is ROS-mediated. Neither antioxidant restored MetHb to baseline, indicating that roughly 55% of the oxidative damage arises from a direct interaction between the drug and the heme microenvironment rather than from exogenous ROS.

The differential scavenger profile—Catalase effective (22% rescue), SOD ineffective, and DFO significantly protective (34% rescue)—maps onto the established chemistry of hemoglobin autoxidation. Superoxide generated during autoxidation dismutates almost instantaneously at physiological pH to H_2_O_2_, which drives downstream Fenton/ferryl chemistry [28]. SOD merely accelerates this dismutation and therefore cannot interrupt the pathway; the protective effect of DFO confirms that loosely bound or free iron is required to catalyze H_2_O_2_-mediated amplification. Thus, Gentamicin-induced MetHb accumulation stems from two parallel routes: direct Fe^2+^ autoxidation triggered by drug-induced perturbation of the heme pocket, and secondary oxidation of neighboring HbA molecules by H_2_O_2_ generated from the initial event.

Clinically, methemoglobinemia compromises tissue oxygenation by abolishing the oxygen-carrying capacity of hemoglobin. These results thus highlight a potential hematological risk of Gentamicin, especially with prolonged use or in individuals with hemoglobinopathies. In vivo validation using animal models should be pursued in future investigations.

### 3.2. Gentamicin Reduces the Oxygen Affinity of HbA

Concurrent with MetHb accumulation, Gentamicin caused a pronounced rightward shift in the oxygen dissociation curve and elevated P50 values. At first glance this is paradoxical: classical cooperativity theory predicts that MetHb formation should relieve allosteric constraints and increase the oxygen affinity of the remaining ferrous subunits. The opposite observation indicates that Gentamicin locks HbA in its low-affinity T-state conformation.

This observation is of particular mechanistic interest. Generally, MetHb formation is accompanied by an increase in oxygen affinity of the residual subunits. When the central heme iron of hemoglobin is oxidized to the ferric state (Fe^3+^), the resulting MetHb loses its intrinsic oxygen-binding capacity, and each 1% increment in MetHb is estimated to decrease the P_50_ by approximately 0.2 mmHg [29].

This unexpected observation is not without precedent. The relationship between MetHb and oxygen affinity is not invariant; rather, it is critically influenced by the mode of oxidation. Notably, Cordone et al. demonstrated that at equivalent MetHb levels, mixed samples exhibit lower oxygen affinity and higher cooperativity than spontaneously oxidized samples, indicating that the context of oxidation profoundly influences the functional outcome [30].

The ODC rightward shift was statistically significant even at 25 μM, a therapeutically relevant plasma concentration. Throughout the concentration range, the Hill coefficient remained unchanged and the Bohr effect was preserved, confirming that the drug does not disrupt subunit cooperativity or proton-sensitive residues. This pattern resembles that of certain NSAIDs (e.g., ibuprofen), which increase P50 at low concentrations without abolishing cooperativity—suggesting that localized binding at the heme pocket entrance is sufficient to bias the allosteric equilibrium without global denaturation.

In practical terms, Gentamicin impairs oxygen delivery through two simultaneous actions: it reduces the number of functional oxygen-binding sites (via MetHb formation), and it weakens the oxygen-binding strength of the sites that remain functional (via T-state stabilization).

The translational relevance is twofold. First, effects initiate at clinically relevant concentrations: at 25 μM (≈12 mg/L, therapeutic peak), P50 shift was significant and MetHb showed a clear upward trend, suggesting effects may be triggered even under routine dosing. Second, effects intensify with increasing concentrations. Aminoglycosides accumulate in the renal cortex to levels several- to tens-fold higher than in plasma, with a half-life of days to weeks. Our gradient data show dose-dependent increases in both MetHb formation and P50 elevation: significant effects were already observed at 150 μM (~6-fold Cmax), and at 900 μM—levels attainable in the renal cortex during long-term therapy or renal impairment. This dose-effect relationship provides experimental evidence for cumulative hematological effects with repeated dosing and offers a mechanistic clue to the clinical coexistence of Gentamicin nephrotoxicity with anemia and tissue hypoxia. However, as these findings are derived from in vitro experiments, their in vivo significance requires further validation.

Furthermore, although Gentamicin is generally considered to lack specific transporters for erythrocyte entry, we observed that it similarly affects erythrocyte P50. This is likely attributable to its exceptionally low equilibrium dissociation constant for HbA (KD = 0.628 μM; Figure 4B), implying that even minimal intracellular accumulation would suffice to account for the observed P50 elevation. More importantly, the erythrocyte membrane remained intact (Figure S4), precluding any possibility of non-specific binding or assay interference resulting from cell rupture. Collectively, these observations suggest that Gentamicin gains intracellular access via non-specific pathways, thereby mediating the observed functional alterations.

### 3.3. Structural and Molecular Basis of Gentamicin-Induced HbA Dysfunction

The function of HbA is intimately associated with its structural characteristics. To elucidate the mechanistic basis of this phenomenon, SPR, UV–Vis spectroscopy, secondary structure analyses, and molecular docking were performed.

Binding between Gentamicin and HbA was characterized by SPR, yielding an equilibrium dissociation constant KD of 6.28 × 10^−7^ M. The KD reflects the degree of receptor–ligand complex dissociation at equilibrium, with a lower KD indicating higher binding affinity. Notably, this value is substantially lower than that of the endogenous HbA effector 2,3-diphosphoglycerate (2,3-DPG; KD = 4.62 × 10^−4^ M), suggesting that Gentamicin can competitively inhibit 2,3-DPG binding. This finding is consistent with a previous report that the fluoroquinolone lomefloxacin competes with 2,3-DPG for its binding site, thereby interfering with the allosteric regulation of hemoglobin [31]. Collectively, these data indicate that Gentamicin binding may compromise normal hemoglobin regulatory function.

The effect of Gentamicin on the secondary structure of HbA was assessed by MMS. The higher-order structure (HOS) of HbA is primarily determined by intramolecular hydrogen bonding between backbone amide and carbonyl groups, a structural arrangement that plays a critical role in directing peptide chain folding and defining overall protein conformation [32]. The global secondary structure of HbA remained essentially unperturbed, and the spectral similarity between HbA and HbA–Gentamicin reached 97.8%, providing a structural rationale for the retained cooperativity (Hill coefficient) and the Bohr effect of HbA.

UV–Vis spectroscopy revealed the corresponding tertiary perturbations. This spectral evolution reflects the spin-state transition from low-spin Fe^2+^–O_2_ to high-spin Fe^3+^. The dissociation—stable secondary structure alongside significant tertiary change—indicates that Gentamicin penetrates deeply enough into the heme pocket to alter iron coordination and porphyrin electronic transitions without attacking the hydrogen-bond network that maintains global folding. Methemoglobin formation is accompanied by a hypochromic shift in the Soret band (decreased absorbance) [33]. Alterations in the Q band (500–600 nm) serve as the most direct and visually diagnostic feature for distinguishing oxyhemoglobin from methemoglobin and constitute the key spectral criterion for detection [34]. In the normal oxygenated (Fe^2+^) state, hemoglobin exhibits a characteristic double-peak profile in the visible region, with absorption maxima at approximately 541 nm and 578 nm. When ferrous iron (Fe^2+^) is oxidized to ferric iron (Fe^3+^) to form methemoglobin, the spin state of the iron ion may undergo a change (to high-spin or mixed-spin), thereby altering the electron cloud distribution and the energy level gaps of the porphyrin ring [35].

Molecular docking was employed to identify the binding site of Gentamicin within HbA. The results revealed that Gentamicin forms hydrogen bonds with β-chain residues His63, His92, and Phe41, a carbon–hydrogen bond with Phe103, and hydrophobic interactions with Val67 and Leu141 (CDOCKER_ENERGY = 27.02 kJ/mol). Molecular dynamics (MD) simulations over a 100 ns trajectory further confirmed stable binding without inducing global unfolding: the protein backbone remained unperturbed, and the ligand maintained a stable conformation throughout the simulation. Ligand binding constrained local residue flexibility and enhanced pocket rigidity, favoring a stable allosteric conformation.

These residues have known functional relevance and collectively provide a structural basis for the oxidative damage and allosteric effects observed in this study.

With respect to the oxidative mechanism, βHis63 is the distal histidine that normally hydrogen-bonds with bound O_2_, stabilizing the oxy complex and suppressing autoxidation. Replacement of this residue with valine accelerates autoxidation by more than two orders of magnitude [36]. Gentamicin binding adjacent to βHis63 displaces this protective gatekeeper, exposing Fe^2+^–O_2_ to solvent and permitting spontaneous oxidation. This structural perturbation accounts for the ~55% of MetHb formation that is refractory to antioxidant rescue.

Regarding the allosteric mechanism, βHis92 is the proximal histidine coordinating the heme iron; drug-induced strain on the Fe–N bond alters the electronic environment of the iron center, favoring a low-affinity T-state geometry. βVal67, also located in the distal pocket, has been shown in mutagenesis studies to reduce oxygen affinity without disrupting cooperativity [37]. Collectively, these atomic contacts stabilize the T-state through synergistic proximal and distal pocket interactions, overwhelming the expected affinity increase from MetHb accumulation. Thus, a single binding event at the heme pocket entrance simultaneously triggers oxidative chemistry (via βHis63) and allosteric chemistry (via βHis92/βVal67).

Furthermore, this binding mode explains why Gentamicin reduces oxygen affinity without affecting the Bohr effect. The Bohr effect reflects the coupling efficiency between H^+^ and O_2_ binding, primarily mediated by key residues including βHis146 and the β-chain N-terminus. Because the Gentamicin binding site involves βHis63, βHis92, βPhe41, βPhe103, βVal67, and βLeu141—none of which are directly involved in proton sensing—the T-/R-state equilibrium is shifted toward the T-state (reducing affinity), while pH-dependent modulation of oxygen binding remains intact. This is consistent with the computational observation that Gentamicin does not interfere with βHis146 or other Bohr effect residues.

In a word, the present study demonstrates that Gentamicin binds to specific sites on the β-chain of HbA and exerts hematological effects through a dual mechanism. Binding at βHis63 disrupts the protective hydrogen-bonding network surrounding Fe^2+^–O_2_, accelerating HbA autoxidation and elevating MetHb levels. The ensuing oxidative cascade is amplified by H_2_O_2_-dependent, iron-mediated chemistry, consistent with the differential rescue profile observed for NAC, GSH, DFO, catalase, and SOD. Concurrently, interactions with βHis92 and βVal67 stabilize the T-state of HbA, reducing oxygen affinity without compromising cooperativity. Collectively, these findings provide a structure-based mechanistic framework linking Gentamicin binding to both oxidative and functional impairment of HbA.

## 4. Materials and Methods

### 4.1. Preparation of HbA and Adult Human RBCs

Venous blood was collected from healthy volunteers via antecubital venipuncture. HbA was purified from whole blood mixed with CPDA-1 (Sigma Aldrich, St Louis, MO, USA) by anion-exchange chromatography and was prepared at a concentration of 5 g/dL, as previously described (Supplementary Materials Section S2) [38].

Whole blood from healthy adult donors was filtered through a leukocyte removal filter to achieve leukoreduction, following the protocol described by Chu et al. [38]. The filtrate was then centrifuged at 800× *g* for 10 min at 4 °C. The plasma and buffy coat were carefully removed and discarded. The packed RBCs were washed three times with ice-cold phosphate-buffered saline (PBS, pH 7.4) and resuspended in PBS to a final hematocrit of 20% (*v*/*v*) for oxygen dissociation assays. All procedures were completed within 24 h of blood collection.

### 4.2. Incubation of the HbA and Gentamicin

HbA and Gentamicin were incubated at pH 7.4. A stock solution of Gentamicin was prepared in phosphate-buffered saline (PBS; Sigma-Aldrich, St. Louis, MO, USA) at a concentration of 100 mM.

### 4.3. Methemoglobin Formation and Scavenger Rescue Assays

HbA (0.09 mM, equivalent to 5 g/dL) was incubated with Gentamicin at final concentrations of 0, 25, 150, or 900 μM. The detection of MetHb was carried out in PBS buffer, pH 7.4, at 37 °C. Absorbance changes in the range of 350–700 nm due to the spontaneous oxidation of Hbs were recorded using a UV-vis spectrometer at 1 h intervals for 8 h. The time-dependent changes in the concentration of MetHb were calculated using the following equations [39]:

MetHb = 2.985 × (OD_630_ − OD_700_) + 0.194 × (OD_576_ − OD_700_) − 0.4023 × (OD_560_ − OD_700_) (1)

Deoxy = 1.373 × (OD_560_ − OD_700_) − 0.747 × (OD_576_ − OD_700_) − 0.737 × (OD_630_ − OD_700_) (2)

OxyHb = 1.013 × (OD_576_ − OD_700_) − 0.3269 × (OD_630_ − OD_700_) − 0.7353 × (OD_560_ − OD_700_) (3)

To elucidate the oxidative pathways underlying Gentamicin-induced MetHb formation, HbA was co-incubated with Gentamicin (900 μM) in the presence of various concentrations of scavengers or chelators. The concentration ranges for each reagent were as follows: N-acetylcysteine (NAC; Sigma-Aldrich, St. Louis, MO, USA), 0–10 mM [40]; reduced glutathione (GSH; Sigma-Aldrich, St. Louis, MO, USA), 0–10 mM [40]; superoxide dismutase (SOD; Sigma-Aldrich, St. Louis, MO, USA), 0–500 U/mL [41]; catalase (Sigma-Aldrich, St. Louis, MO, USA), 0–500 U/mL; deferoxamine (DFO; Sigma-Aldrich, St. Louis, MO, USA), 0–200 μM [42]; and ethylenediaminetetraacetic acid (EDTA; Sigma-Aldrich, St. Louis, MO, USA), 0–200 μM [43]. Each scavenger or chelator was also tested alone (without Gentamicin) as a control. MetHb content was determined at the 8 h endpoint. All experiments were performed in triplicate.

### 4.4. Oxygen Dissociation Curve (ODC)

HbA and the HbA–Gentamicin were each diluted in 4 mL of BLOODOX-Solution buffer (pH 7.4) and subsequently combined with 20 μL of 20% bovine serum albumin (Sigma-Aldrich, St. Louis, MO, USA) and 20 μL of anti-foaming agent (Sigma-Aldrich, St. Louis, MO, USA) [38]. Samples were then analyzed using a BLOODOX-2018 analyzer (Softron Biotechnology, Beijing, China), and ODC, P_50_ values and Hill coefficient were recorded [38].

Adult human RBCs were processed and analyzed in the same manner for ODC, P_50_, and Hill coefficient determinations. Adult human RBC integrity was verified by measuring free hemoglobin and LDH release; detailed procedures are provided in the Supplementary Materials (Section S5).

The Bohr effect was evaluated for both HbA and the HbA–Gentamicin complex employing the methodology delineated above, with the exception that the BLOODOX-Solution buffer was adjusted to pH values of 7.2, 7.4, and 7.6. The △SO_2_ value of HbA in simulated plain and plateau environments (6000 m) after binding Gentamicin was calculated. The formula was as follows:

△SO_2(100–40 mmHg)_ = SO_2(100 mmHg, pH7.6)_ − SO_2(40 mmHg, pH7.2)_ (4)

△SO_2(60–30 mmHg)_ = SO_2(60 mmHg, pH7.6)_ − SO_2(30 mmHg, pH7.2)_ (5)

where △SO_2(100–40 mmHg)_ represents the theoretical oxygenrelease capacity of HbA in the plain environment and △SO_2(60–30 mmHg)_ represents the theoretical oxygen-release capacity of HbA in the plateau environment; SO_2(100 mmHg, pH7.6)_ and SO_2(60 mmHg, pH7.6)_ represents the HbA oxygen saturation at pH 7.6 and an oxygen partial pressure of 100 mmHg and 60 mmHg, which simulates the environment in the lung under plain and plateau conditions; SO_2(40 mmHg, pH7.2)_ and SO_2(30 mmHg, pH7.2)_ represents the HbA oxygen saturation at pH 7.2 and an oxygen partial pressure of 40 mmHg and 30 mmHg.

### 4.5. Oxygen Dissociation Assay (ODA)

ODA is a screening assay based on spectral changes in HbA during deoxygenation to represent the oxygen release of HbA [44]. HbA (3 µM) and HbA–Gentamicin (1:10 molar ratio) were each diluted in DPBS adjusted to pH 7.0, 7.2, 7.4, 7.6, and 8.0, and oxygenated with air for 50 min (10 cycles) at 37 °C in 96-well optically transparent plates. Deoxygenation was achieved by N_2_ purging at 20 L/min in a UV/Vis spectrometer (Omega, BMG Labtech, Ortenberg, Germany). Spectra (350–700 nm, 1 nm resolution) were acquired every 5 min per cycle. OxyHb% was calculated as Equations (1)–(3) and (6):

OxyHb% = Oxy-Hb/(Met-Hb + Deoxy + Oxy-Hb)(6)

### 4.6. Surface Plasmon Resonance Technology (SPR)

The interaction between Gentamicin (TargetMol, Wellesley Hills, MA, USA) and HbA was assessed by SPR using a Nicoya SPR instrument (Nicoya Life Science, Inc., Kitchener, ON, Canada) equipped with a carboxyl sensor chip [20]. HbA was diluted to 5 μg/mL in immobilization buffer (10 mM sodium acetate, pH 4.5). Gentamicin was serially diluted in PBS to concentrations of 0, 0.125, 0.25, 0.5, 1.0, 2.0, 4.0 μM and 8.0 μM and injected at a flow rate of 20 μL/min, with association monitored for 240 s followed by dissociation for 480 s. Both phases were conducted in running buffer consisting of 1× HEPES (10 mM HEPES, 150 mM NaCl, 3 mM EDTA, 0.005% Tween-20) adjusted to pH 7.4. Kinetic parameters were derived using Trace Drawer software v1.8.1 (Ridgeview Instruments AB, Uppsala, Sweden) [45,46].

Raw sensorgrams were processed by subtracting the reference channel signal and the running buffer blank cycle signal, followed by smoothing with a Gaussian filter (σ = 2.5) to reduce high-frequency noise.

Kinetic parameters were obtained by global fitting to a 1:1 Langmuir model using Trace Drawer software. To assess inter-assay reproducibility, three independent experiments were performed on separate days using freshly prepared Gentamicin dilutions and sensor chips. Fit quality was assessed by reduced chi-square, RMSE, and parameter uniqueness (U-values).

### 4.7. Microfluidic Modulated Spectroscopy (MMS)

Secondary structures of HbA and the HbA–Gentamicin complex were assessed by MMS as previously described [20]. Spectra were acquired on an AQS3^®^pro MMS system (RedShiftBio, Burlington, MA, USA). Original absolute absorbance spectra were recorded over 1701–1598 cm^−1^ and converted to second-derivative plots. Characteristic peaks corresponding to secondary structural elements were identified, and higher-order structure (HOS) components—alpha-helix, beta-sheet, unordered, and beta-turn—were quantified. Spectral processing and data analysis were carried out using AQS3^®^ delta control software(RedShift BioAnalytics; accessed on 10 September 2024).

### 4.8. Molecular Docking

Molecular docking was performed with Discovery Studio 2019 [47]. The three-dimensional structures of HbA and Gentamicin were retrieved from the Protein Data Bank (PDB ID: 1B86) and PubChem (CID: 3467), respectively. The HbA structure was prepared at pH 7.4. Ionizable residues were assigned using PDB2PQR v3.7.1, and the protonation states of histidine residues were manually verified. Gentamicin contains multiple amino groups (pKa > 8.5) and is fully protonated at pH 7.4. Its predominant protonated microstate was generated using OpenBabel v2.3.2 (with the −p 7.4 flag) or Chemicalize, and the ligand was subsequently processed with prepare_ligand4.py (MGLTools v1.5.6) to obtain the PDBQT file.

For binding site selection, the docking grid was centered on the co-crystallized ligand 2,3-DPG (PDB: 1B86) located in the central water cavity. The grid center was set to (x = −14.4, y = 5.8, z = 19.2) Å, with box dimensions of 15.8 Å × 18.0 Å × 15.4 Å and a spacing of 0.375 Å, covering the entire allosteric pocket.

To validate the docking protocol, the co-crystallized 2,3-DPG was extracted and re-docked into its binding site using AutoDock Vina 1.2.5 with the same grid parameters as described above. Exhaustiveness was set to 64, and a maximum of nine output conformations were generated. The RMSD of non-hydrogen atoms between the best docked pose and the original crystal conformation was calculated to assess reproducibility.

The resulting structure was designated as the receptor for the CDOCKER protocol. Ten duplicate conformers of Gentamicin were generated and centered on the defined binding site. Each was subjected to molecular docking-based simulated annealing and subsequently refined by energy minimization, yielding 10 docked poses. The pose achieving the highest docking score was selected as the best-docked conformation, and the conformer with the lowest binding free energy was retained for further analysis.

### 4.9. Molecular Dynamics Simulations

A 100 ns all-atom MD simulation of the Gentamicin–hemoglobin complex was performed with GROMACS 2025, using AMBER14SB for the protein and GAFF2 for the ligand. The complex was solvated in a TIP3P water box (1.2 nm padding) with 0.15 M NaCl. Following energy minimization, the system was equilibrated under NVT (100 ps) and NPT (100 ps, 310 K, 1 bar) ensembles. The production run (100 ns, 2 fs time step) used LINCS constraints and PME electrostatics (cutoff 1.2 nm); frames were saved every 100 ps. The trajectory was analyzed with GROMACS (v2024.3) tools for RMSD, RMSF, Rg, SASA, and protein–ligand hydrogen bonds. A free energy landscape was built using RMSD and Rg as reaction coordinates. The equilibrated complex backbone was aligned to the 2,3-BPG–hemoglobin crystal structure (PDB: 1B86) in PyMOL v2.5.2 (Cα alignment), and the Gentamicin–2,3-BPG centroid distance was measured. MM-PBSA binding free energy was computed over the 40–100 ns stable segment, neglecting entropy.

## 5. Conclusions

In summary, this study identifies HbA as a novel direct target of Gentamicin in vitro. The drug induces a distinctive functional signature—elevated methemoglobin accompanied by reduced oxygen affinity—mediated through direct binding and tertiary structural changes, likely arising from its pro-oxidant chemistry. These findings open a new avenue for understanding the full toxicological profile of this widely used antibiotic.

From a toxicological perspective, these observations suggest that the risk profile of Gentamicin may extend beyond organ-specific damage to encompass impaired oxygen delivery. Whether such biochemical changes translate into impaired oxygen delivery in patients remains to be established in vivo.

## 6. Limitations and Future Perspectives

First, although the in vitro system employed in this study allows for effective control of experimental variables, it does not fully recapitulate the complex hematological microenvironment. Plasma proteins may sequester Gentamicin and thereby reduce its free concentration, while endogenous antioxidant systems may modulate the redox state of hemoglobin. Accordingly, the observed biochemical effects should be interpreted as molecular and cellular phenomena rather than as established clinical toxicities. Second, the molecular docking and molecular dynamics simulations are computational predictions, and the identified interaction sites require further experimental validation. Third, intracellular gentamicin concentrations were not directly measured in erythrocyte experiments, and the specific pathway of drug entry into erythrocytes remains to be determined.

For future investigations, site-directed mutagenesis could be used to experimentally validate the predicted binding sites; radioisotope tracing or fluorescence imaging could enable quantitative analysis of intracellular drug accumulation in erythrocytes; and key experiments could be replicated in systems containing physiological concentrations of plasma proteins. Additionally, the translational relevance of our findings should be evaluated in clinical samples and animal models.

## Figures and Tables

**Table 1:** Dynamics and affinity parameters of Gentamicin binding to HbA.

Parameter	Value ± SE	CV (%)
Ka (×10^4^ M^−1^·s^−1^)	1.009 ± 0.065	6.4
Kd (×10^−3^ s^−1^)	6.31 ± 0.21	3.3
KD (×10^−7^ M)	6.28 ± 0.60	9.6

**Table 2:** The MM/PBSA Binding free energy (kcal/mol) for the HbA–Gentamicin complex.

Energy Component	Average	SEM
ΔE_vdW	−23.45	0.54
ΔE_ele	−60.66	4.23
ΔE_PB	18.08	3.98
ΔE_np	−3.82	0.05
ΔG_gas a	−84.11	4.06
ΔG_solv b	14.26	3.99
ΔG_bind	−69.85	0.65

## Data Availability

The original contributions presented in this study are included in the article. Further inquiries can be directed to the corresponding authors.

## Supplementary Materials

The following supplementary materials can be downloaded at https://www.mdpi.com/article/10.3390/ijms27177760/s1.

## Abbreviations

The following abbreviations are used in this manuscript:

MetHb	Methemoglobin
Adult Hemoglobin	HbA
UV-Vis	Ultraviolet visible
ROS	Reactive oxygen species
ODA	Oxygen Dissociation Assay
ODC	Oxygen Dissociation Curve
SPR	Surface Plasmon Resonance Technology
MMS	Microfluidic Modulated Spectroscopy
HOS	Higher-order structure
