# Supplementary material for: Gentamicin Targeting Human Hemoglobin Induces Methemoglobin Formation and Decreases Oxygen Affinity: A Molecular Mechanism of Hematologic Toxicity
Source: Int J Mol Sci. 2026 Aug 29;27(17):7760. doi: 10.3390/ijms27177760 (PMC13566497; doi:10.3390/ijms27177760)
Supplement: Supplementary file 1 [file ijms-27-07760-s001.zip › ijms-4432629-supplementary.pdf]

S1. Theoretical oxygen-release capacity

Theoretical oxygen-release capacity refers to the amount of oxygen that hemoglobin can theoretically release during transport from the lungs to tissue cells, and is quantified by the oxygen saturation difference (ΔSO_2) between the lungs and tissue cells. The calculation integrates the Bohr effect and the oxygen partial pressure gradient. For the Bohr effect, pH 7.6 and pH 7.2 are used to simulate the acid–base conditions of the lung and tissue environments, respectively. For the oxygen partial pressure gradient, two scenarios are defined: a plain condition (approximately 100 mmHg → 40 mmHg) and a high-altitude condition (approximately 60 mmHg → 30 mmHg). The schematic diagram is shown in Figure S1

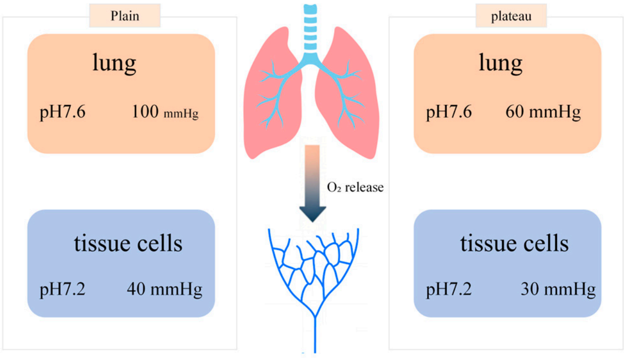

Figure S1.The schematic diagram of The "theoretical oxygen-release capacity"

S2. Human erythrocytes were washed, lysed, and centrifuged. The resulting hemolysate was filtered, dialyzed, and purified using an AKTA Pure system equipped with a Q Sepharose HP anion-exchange chromatography column. The target HbA fractions were collected, concentrated by ultrafiltration (10 kDa molecular weight cutoff), and stored at –80 °C. Purity was assessed by SDS-PAGE (10 µg protein per lane, stained with Coomassie Brilliant Blue) combined with densitometric scanning. The final purity of the HbA preparation was determined to be ≥99%.

S3.SPR

Raw sensorgrams were processed by subtracting the reference channel signal and the running buffer blank cycle signal. A mild Gaussian filter ($\sigma = 2.5$) was then applied to suppress high-frequency noise. To clearly demonstrate the effect of this smoothing step, the original figures have been replaced with overlaid comparisons of the unsmoothed and smoothed data, and the completely unsmoothed raw sensorgrams are provided in the Figure S2. Kinetic analysis was performed by globally fitting the sensorgrams at three concentrations to a 1:1 Langmuir model. Owing to limited sample availability, only one independent experiment could be performed; therefore, the standard errors and 95% confidence intervals of the kinetic parameters were estimated from the covariance matrix of the fit. The quality of the fit was assessed by the root-mean-square error of residuals (RMSE), reduced chi-square (χ^2/ν), parameter uniqueness (U-values).

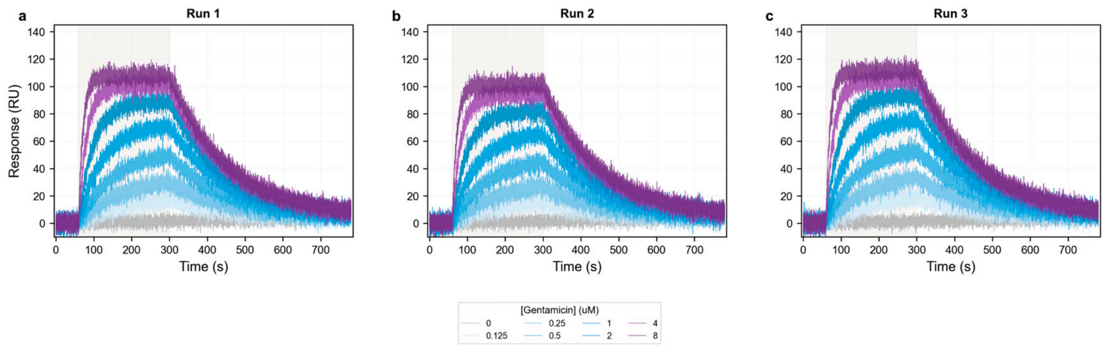

Figure S2

Figure S2. Raw SPR sensorgrams from three independent runs (a-c).

Table S1. Fitting statistics for three independent SPR runs.

.

Parameter	Run 1	Run 2	Run 3
Weighted chi2	125,800	128,400	131,200
Total data points	62,400	62,400	62,400
Fitted parameters	4	4	4
Degrees of freedom	62,396	62,396	62,396
Reduced chi2 (chi2/df)	2.02	2.06	2.10
RMSE (RU)	2.4	2.6	2.7
U-value (Rmax)	4.8	4.3	5.1
U-value (ka)	9.5	8.9	10.2
U-value (kd)	11.0	10.5	11.8
U-value (KD)	5.8	5.3	6.2

S4. Gentamicin Reduces the Oxygen-Carrying Capacity of HbA and Rat's RBCs

ODC were recorded. As shown in Figure S3. For rat RBCs, the P50 of the rat RBCs was 23.09 ± 1.61 mmHg, and Gentamicin elevated it to 34.80 ± 1.76 mmHg (vs. rat RBCs, $P < 0.05$). These data demonstrate that Gentamicin decreased the oxygen affinity of both HbA and rat RBCs. In addition, Gentamicin did not significantly affect the Hill coefficient of both HbA and rat RBCs.

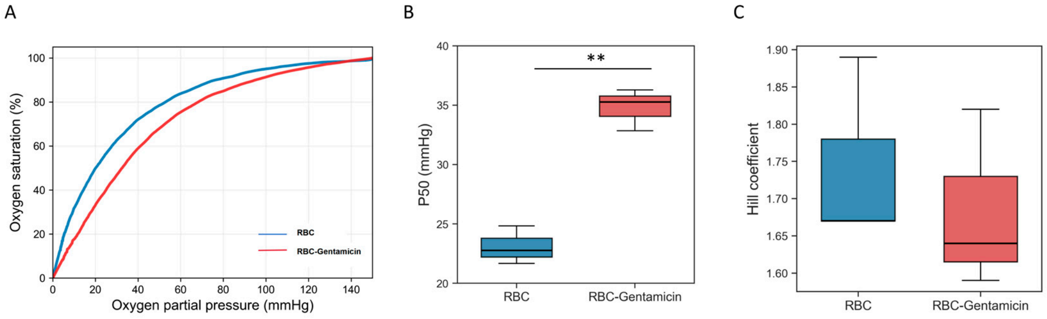

Figure S3. Effect of Gentamicin on oxygen affinity of rat RBCs. (A) The ODC of RBC and RBC–Gentamicin (1:10). (B) P50 value of RBC and RBC–Gentamicin. Gentamicin significantly

increased the P50 value of HbA. (C)Hill coefficient of RBC and RBC–Gentamicin. Error bars represent the standard deviation (SD). ** indicates $p < 0.01$.

S5. Red Blood Cell Integrity Assessment

To exclude hemolysis during ODC measurements, adult human RBC suspensions were incubated with Gentamicin under the same conditions as for ODC assays (PBS pH 7.4, 37 °C). After incubation, samples were centrifuged at 800 × g for 10 min at 4 °C, and the supernatant was collected.Free hemoglobin was measured using Drabkin's reagent (Sigma-Aldrich, St. Louis, MO, USA): 100 µL supernatant was mixed with 1 mL Drabkin's reagent, incubated for 5 min at room temperature, and absorbance was read at 540 nm. LDH activity was determined using an LDH Cytotoxicity Detection Kit (Roche Diagnostics, Mannheim, Germany): 100 µL supernatant was mixed with 100 µL reaction mixture, incubated for 30 min in the dark, and absorbance was read at 490 nm. Hemolysis (%) and LDH release (%) were calculated relative to complete lysis induced by 1% Triton X-100. All assays were performed in triplicate.

As shown in Figure S4, free hemoglobin concentrations in the supernatant across all gentamicin concentrations (0–900 µM) were consistently below 2.5 mg/dL, and the hemolysis rates were below 0.5%, with no significant differences compared to the control group ($p > 0.05$). Similarly, LDH release rates showed no statistical differences among the groups ($p > 0.05$), remaining below 2%. Although slight concentration-dependent increases were observed at 900 µM for free hemoglobin (2.19 ± 0.41 mg/dL), hemolysis rate (0.43 ± 0.15%), and LDH release (1.67 ± 0.52%), these differences were not statistically significant. In contrast, the positive control (1% Triton X-100) induced complete lysis (582.4 ± 24.7 mg/dL free hemoglobin; 100% hemolysis; 100% LDH release), thereby validating the sensitivity of the assay. Collectively, these results indicate that RBCs remained intact without obvious lysis after incubation with gentamicin, ruling out false-positive results caused by hemolysis.

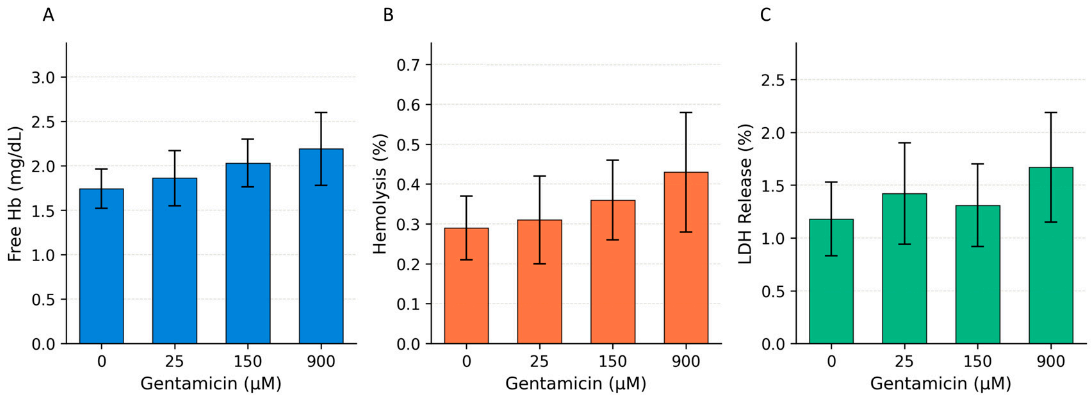

Figure S4. Effect of Gentamicin on erythrocyte membrane integrity. (A) Free hemoglobin in the supernatant. (B) Hemolysis rate calculated relative to complete lysis with 1% Triton X-100 (defined as 100%). (C) LDH release rate relative to the Triton X-100 control (defined as 100%).
